# How do GPs identify a need for palliative care in their patients? An interview study

**DOI:** 10.1186/1471-2296-14-42

**Published:** 2013-03-25

**Authors:** Susanne JJ Claessen, Anneke L Francke, Yvonne Engels, Luc Deliens

**Affiliations:** 1 VU University medical center (VUmc), EMGO Institute for Health and Care Research, Department of Public and Occupational Health & Expertise Center Palliative Care VUmc, Amsterdam, the Netherlands; 2 NIVEL, Netherlands Institute for Health Services Research, Utrecht, the Netherlands; 3 Radboud University Nijmegen Medical Centre, Department of Anesthesiology, Pain and Palliative Medicine, Nijmegen, the Netherlands; 4 Ghent University & Vrije Universiteit Brussel, End-of-life Care Research Group, Brussels, Belgium

**Keywords:** General practice, Primary care, Palliative care, Qualitative research, End-of-life care

## Abstract

**Background:**

Little is known about how GPs determine whether and when patients need palliative care. Little research has been done regarding the assumption underpinning Lynn and Adamson’s model that palliative care may start early in the course of the disease. This study was conducted to explore how GPs identify a need for palliative care in patients.

**Methods:**

A qualitative interview study was performed among 20 GPs in the Netherlands.

**Results:**

GPs reported that a combination of several signals, often subtle and not explicit, made them identify a need for palliative care: signals from patients (increasing care dependency and not recuperating after intercurrent diseases) and signals from relatives or reports from medical specialists. GPs reported differences in how they identified a need for palliative care in cancer patients versus those with other diseases. In cancer patients, the need for palliative care was often relatively clear because of a relatively strict demarcation between the curative and palliative phase. However, in patients with e.g. COPD or in the very old, GPs' awareness of palliative care needs often arises gradually, relatively late in the disease trajectory.

GPs consider the diagnosis of a life-threatening illness as a key point in the disease trajectory. However, this does not automatically mean that a patient needs palliative care at that point.

**Conclusions:**

GPs recognize a need for palliative care on the basis of various signals. They do not support the idea underlying Lynn and Adamson’s model that palliative care always starts early in the course of the disease.

## Background

Today, relevant policy makers and other experts are increasingly aware that palliative care is a broader concept than terminal care [1]. In 2003, a 'model' of palliative care was introduced by Lynn and Adamson [2] that underlined the necessity of an early start of palliative care. Initially, curative, disease-modifying and life-prolonging treatments may be given alongside palliative treatments, with a gradual shift to an emphasis on palliation. However, a previous study in the Netherlands indicated that in practice the shift from curative and life-prolonging treatments towards palliation often occurs at a late stage in the disease trajectory [3], suggesting that sometimes GPs or other relevant professionals only recognize patients' need for palliative care late in the disease trajectory. However, timely recognition may be important in order to enable advance care planning and prevent crisis situations and unnecessary care transitions or hospital admissions.

Nowadays there is also an increasing awareness that palliative care is not only for patients with incurable cancer but also for patients with COPD, heart failure or dementia, for instance. GPs' recognition of a need for palliative care may be different for cancer patients than for patients with other diseases since disease trajectories vary [4,5]. Lynn and Adamson have described three common disease trajectories [2]. First, there is a trajectory often seen in cancer patients, characterized by a relatively short period of evident decline, a rather clear demarcation between the curative and palliative stages and a foreseen death. In contrast, the disease trajectory of patients with heart failure or COPD is often characterized by long-term limitations, intermittent exacerbations and remissions, resulting in a relatively sudden death. In older people there is often a prolonged gradual deterioration towards death. Various disease trajectories can therefore be distinguished that may be related to variations in the timing and nature of the recognition of a patient's need for palliative care. However, few studies have been conducted to date on GPs’ recognition of their patients’ need for palliative care [6].

The scarcity of research literature on GPs’ recognition of a need for palliative care is remarkable, since GPs often have a long 'history' with the patient and family and also play a pivotal role in palliative care. In the Netherlands, for example, palliative care is mainly provided by ‘general’ health care professionals, like the GP and home care professionals, to guarantee that this care is accessible for everyone who is in need of it. Hence, palliative care is integrated into the regular healthcare system, in which GPs function as gatekeepers for specialized care services and as family doctors.

Considering GPs’ pivotal role in palliative care this qualitative study was conducted to explore how GPs recognize a need for palliative care in various patient groups.

The specific research questions addressed in this paper are:

1. How do GPs recognize a need for palliative care?

2. Does this recognition generally arise in the diagnosis stage or in later stages of the disease trajectory?

3. Are there any differences in the timing and nature of GPs' recognition of the need for palliative care between cancer patients and non-cancer patients?

## Methods

### Sample and recruitment

In the Netherlands, all GPs have had mandatory postgraduate GP training after their basic medical training. GPs were recruited in several ways. First, GPs who had participated in a previous survey about palliative care in general practice had been asked whether they were also willing to be interviewed. Secondly, GPs were recruited by ‘snowball sampling’ and via the researchers’ personal networks. Purposive sampling was conducted to guarantee variation in background characteristics such as age, gender, experiences with palliative care, degree of urbanization and kind of practice.

Recruitment of GPs stopped after data saturation was reached (i.e. no additional themes relevant for answering the research questions emerged during additional interviews and subsequent data analysis).

### Ethical procedures

According to Dutch law, approval by a medical ethics committee is not needed for non-experimental interview data involving competent adults. The anonymity of the GPs was strictly preserved throughout the data entry and analysis process.

All GPs were given verbal and written information about the aim and scope of the interviews and all GPs gave written informed consent.

### Interviews

All interviews were conducted by the first author (SC), who was supported and accompanied in the first two interviews by the second author (AF). The interviews were performed at the GP’s practice or home, at an appropriate moment for the GP.

The interview questions were compiled by the four authors (one medical doctor/PhD student, two professors and one assistant professor in palliative care) and refined on the basis of recommendations by the steering committee (two GPs and one professor in communication and education of family medicine). The interviews were semi-structured using a topic list with open interview questions (see the Additional file 1).

The interviews were audio taped, transcribed verbatim and rendered anonymous.

### Analysis

As usual in qualitative research, data analysis started after the initial interviews were conducted as part of a cyclical process of data collection – data analysis – new data collection, et cetera. Each interview was transcribed literally and read and reread, keeping the research questions in mind. In accordance with the guidelines for qualitative analysis of Dierckx de Casterlé [7], the first author (SC) subsequently made narrative interview reports describing the main findings from each interview. The second author (ALF) did the same for half of the interviews. The pairs of narrative interview reports for the same interview were compared and discussed in relation to the research questions. In addition, the first author (SC) systematically coded the interview transcripts, initially by ‘open coding’, which means identifying, naming and categorizing relevant themes found in the transcripts. This was followed by ‘axial coding’. Axial coding involves looking for connections between categories of codes. Ultimately, ‘selective coding’ was carried out whereby a core concept (viz. subtle signals from patients) was determined [8]. The coding process was supported by the Atlas.ti program [9]. This software program sorts relevant fragments and links these to other fragments with the same codes within a single interview and in other interviews. The code book was discussed and agreed upon with the second author (AF). Results from the interim and final analyses were reported and discussed during meetings with the other co-authors.

## Results

### Participating GPs

We conducted qualitative interviews with ten male and ten female GPs between spring 2011 and spring 2012. Mean interview duration was about 50 minutes. Mean age of the GPs was 48 years (range of 30–62). GPs on average worked 37 hours a week (range 20–60). There was a mix of GPs working in group practices (n= 17) and in one-person practices (n= 3). The practice location areas ranged from highly urbanized regions (n= 11), slightly urbanized (n= 5) and less urban/rural regions (n= 4). An overview of the participants’ characteristics is presented in Table 1.

**Table 1:** Overview of participants’ characteristics

**GP number**	**Gender**	**Age (range)**	**Working hours per week**	**Practice location area**
1	Female	50-55	40	highly urbanized
2	Female	56-60	24	slightly urbanized
3	Male	50-55	40	highly urbanized
4	Female	40-45	28	highly urbanized
5	Female	60-65	36	highly urbanized
6	Female	56-60	60	less urban/rural
7	Male	50-55	50	highly urbanized
8	Male	60-65	32	highly urbanized
9	Male	30-35	40	slightly urbanized
10	Male	30-35	42	slightly urbanized
11	Male	50-55	40	highly urbanized
12	Female	40-45	36	slightly urbanized
13	Male	56-60	40	highly urbanized
14	Female	36-40	30	slightly urbanized
15	Male	36-40	40	highly urbanized
16	Female	40-45	20	less urban/rural
17	Male	50-55	30	highly urbanized
18	Male	60-65	55	less urban/rural
19	Female	40-45	30	less urban/rural
20	Female	30-35	27	highly urbanized

### Interview results

#### GPs’ views on the start of palliative care

GPs mainly associate palliative care with a relatively circumscribed period of a few weeks or months in which incurably ill people need a great deal of physical, psychosocial and spiritual care. Some GPs add that they realize palliative care may start years before death in the case of certain chronic diseases such as COPD or heart failure. However, the immediate association is still with a limited period at the end of life in which the patients (and often their close relatives) make increasing demands on the GP, care needs become more intensive, and contacts become more frequent.

(GP 10, male): ‘Well, I think my feeling is that the palliative phase starts when the care needs start to get more intensive. So when you notice that contact isn’t just once every three months… just asking hey, how’s it going, if that shifts to more intensive care, where your help is demanded on a more regular basis or where you feel perhaps I should pop in more often to keep an eye on things, to my mind that is a kind of start to the palliative phase.’

While GPs do see palliative care as more than just terminal care, and some are clearly familiar with Lynn and Adamson’s model (see introduction), that is not sufficient reason for them to start using the term palliative care right from the moment of diagnosis. Nevertheless, GPs do see the diagnosis phase as an important phase, and they offer support to the patient and relatives to cope with the diagnosis. However, GPs feel there is little to be gained from seeing this as the start of palliative care, especially as many people with cancer, for example, will eventually recover. Patients are often not yet dependent on healthcare in the early stage of a life-threatening disease, and in such situations GPs feel it does not make sense to talk of ‘palliative care’.

(GP 19, female) ‘Well yes… if it really is about people with COPD or people with heart failure and so on - and I think these are mainly the people we are talking about, or people with muscular conditions - even though you know someone is eventually going to die from this there is a long period before dying, you can say it’s really a chronic disease. You know they will eventually die from it. I find it difficult to say whether you should call that entire period palliative care. Well… as I said, well we discussed it earlier when I said that I’m not thinking about that, I don’t start offering different care because it’s suddenly called palliative care or chronic care. Because you always try just to make things as comfortable as possible for the patient. So perhaps the label applied to it is just not so important. That's my feeling… why should you call it that?’

It is often not the diagnosis but a combination of generally subtle signals that prompts GPs to consider palliative care needs. These signs come initially from the patient, frequently in combination with reports by other care professionals and close relatives. We explain the different signs below.

### Subtle signals given by patient

#### Changes in self-care abilities and care dependency

The recognition of a need for palliative care is often a gradual process steered by a combination of signals. One key signal is a reduction in a patient’s self-care ability, in the sense that it costs a patient an increasing amount of effort to look after himself. Often, a patient also becomes increasingly bedridden and dependent on care by relatives and professionals. The care needs addressed to the GP also become more frequent and more intense. A patient who always used to come to the GP practice may suddenly request a home visit. Two GPs describe increasing healthcare demands in the interview excerpts below.

(GP 3, male): ‘It starts when the care need increases.’

Interviewer: ‘Could you explain that?’ ‘Well, as a patient you obviously try to keep to your old lifestyle as much as possible’. And if you have to give that up and ask for help because you can no longer manage to eat or sleep or because of the pain, that is the point when you can give a patient support.’

(GP 1, female): ‘Yes, there are different kinds of palliative care. And the real palliative care is the final stage as it were, because then more care is needed from the people around them or from outside. And if someone becomes progressively dependent on others. I think that this… is so for various activities of daily living, that they can no longer do themselves.’

#### Not recuperating

Not recuperating fully after an additional condition, such as bladder infection, is also a signal for GPs that they need to monitor this patient particularly closely. Normally people should be back to normal, but the anticipated recovery never happens or is only partial, and this is a signal for GPs that their patients are ‘sliding’ into the palliative phase.

(GP 4, female): ‘These are people who sometimes don’t recuperate after a particular condition as you expect - hey, I treated that bladder inflammation and… so it is partly a case of expectations and knowing that if I treat that condition they should recuperate after a certain period. Then someone should be back to normal, but look, they’re not. And that’s often the start.’

#### Social changes

Social changes are also a signal for GPs that there is a need for palliative care, for example if people become withdrawn, less focused on contacts beyond their close relatives and no longer get pleasure from hobbies or going out. Life becomes more ‘existential’ in the sense that they concentrate on the people and things that are most important to them, and focus on closure and taking leave of life.

### Signals from close relatives

GPs also often get signals from close relatives. A partner or other close family member will often report deterioration in the patient’s situation or say that the burden of care is becoming very heavy and that they need to discuss matters.

(GP 9, male): ‘Yes, or the family carers for example. They may well be the biggest source. You see the family carer, who often ends up dealing with most of the demands for help and care, and they say, well, I’m finding it a bit too much, I’m finding the going too tough, I need someone I can discuss things with now and then. And you see the family carers are often just as pleased with a GP who visits regularly as the patients themselves are. So they also play an important part.’

### Reports by other professionals

Another signal for GPs that a patient may require palliative care is a message from the medical specialist that cure is not, or no longer, a possibility. This is also the point at which the medical specialist refers the patient back to their GP, who once again becomes the primary treating physician.

Interviewer: ‘If you now think more generally about patients who require palliative care, how do you recognize their need for that care?’

(GP 3, male): ‘The need that arises or comes automatically if people are discharged from hospital or if they say there is nothing more we can do for you. That’s really when it begins…’

The home care organization may also sound the alarm. For instance, the district nurse may phone the GP to say there are problems with the patient, the family is overburdened and something needs to be done. A signal like that in combination with other signals alerts a GP and makes them feel there is a need for palliative care.

### Differences in recognition of palliative needs for different conditions

GPs mention differences in the recognition of a need for palliative care depending on the conditions people are suffering from.

There is often a relatively clear demarcation between the curative and palliative phases for cancer patients, as a medical specialist will say curative treatment is no longer possible.

An additional factor is that the diagnosis of cancer is often experienced as a ‘bombshell' by the patient, their close relatives and sometimes even the GP. Even if GPs feel that palliative care does not automatically start with the diagnosis, in the case of cancer everyone is aware of the real chance that the patient will die. As a result, the option of palliative care is on top of mind at an earlier stage than is the case for many chronic diseases.

(GP 10, male): ‘Usually it’s the case that as soon as they have had the diagnosis, people start to think this could well mean I die and I am still so young. Or fine, I’ve had a good life, I have to die of something… but at any rate they’ll be aware they might die when they get the diagnosis. So that’s a totally different process. So I feel these people often… of course they sometimes also get the shock of seeming to have completely recovered and then they collapse again. But of course often people… the attempts at a cure don’t work out and they gradually get reconciled to the idea. And then it’s a very clear process, then they are thinking about it. And for you as a doctor… these are very intensive things… but I do think there is a certain clarity.’

GPs explain that it is often more difficult to predict how other chronic, ultimately terminal conditions will progress compared with cancer. People with COPD, heart failure or Parkinsonism are often still being treated by a medical specialist in the final stages. They do not have a clear point where the medical specialist says there are no more treatment options. In the excerpt below, a GP explains how he often gradually becomes aware of a need for palliative care.

(GP 9, male): ‘Well, as I said, it is relatively somewhat easier with cancer patients because then you often get a message from the hospital that the curative treatment has failed, so then you know that from that moment on these people are officially palliative. Although certainly at first we often don’t get that involved. And in the case of COPD or heart failure and so on it’s more of a question of noticing at a particular point, well, we seem to be doing palliative things, without it being an explicit decision, but more that you… yes, there comes a point where the policy gradually changes… and there comes a point when you realize, yes, we really are giving palliative care.’

GP also frequently have the feeling with very old people that the patients often shift slowly and gradually to ‘a palliative process’. They describe this as a natural development, the ‘circle of life’. A GP explains:

(GP 8, male): ‘But after they get to 85 or so, lots of people are finished with life, or no longer see it as a real… yes, as a diversion that they are going to die or that it is coming to an end. They're finished with life, so dying is a very natural thing. And then you don’t have so much of an explicit feeling of now we are going to opt for palliative care.’

## Discussion

### Summary of main findings

GPs recognize a need for palliative care on the basis of various, often subtle signals. An important signal is a rise in a patient’s care dependency, in the sense of an increasing need for help in daily functioning and a growing reliance on family members and professionals. In addition, not recovering after intercurrent diseases may be a signal that there is a need for palliative care. These signals are often accompanied by reports by family members, home care professionals and medical specialists. Thus, GPs also take into account the patients’ context and environment when recognizing palliative care needs.

Remarkably, GPs did not mention psychosocial or spiritual problems among the initial signals that a patient was in need of palliative care, although they did consider psychosocial and spiritual support as elements of palliative care.

We found differences between recognizing palliative care needs among patients with cancer, patients with other chronic diseases and the very old, who often deteriorate slowly. In cancer patients, the demarcation between the curative and palliative phases is often relatively clear for GPs. In the case of these patients, the medical specialist informs the GP when curative treatments are not an option or have been stopped. Then the patient is referred from the medical specialist to the GP and a palliative care policy is started. This makes it relatively easy for GPs to demarcate the need for palliative care. However, in patients with chronic diseases such as COPD and heart failure, the course of the disease is often less predictable and patients continue to be treated both by a medical specialist in the hospital and their GP. Consequently, recognition of the need for palliative care often arises gradually. Furthermore, in the case of the ‘oldest old’ where physical and cognitive functions often deteriorate steadily, GPs experience the gradual decline as the normal course of life. Therefore, recognition of the need for palliative care also arises gradually. This finding is in line with the research of Shipman et al. [10]. According to this research, prominent concerns included difficulties with prognosis and the availability of adequate support for patients with advanced non-malignant disease.

Currently, experts like Lynn and Adamson consider the palliative phase as a care continuum which starts early in the course of a life-threatening illness [1,2,11,12]. Dutch GPs also consider the diagnosis stage as an important stage in the patient’s disease trajectory. However, they do not necessarily see the diagnosis as the starting point for palliative care. Although GPs may give support at the time of the diagnosis, they do not call it palliative care as long as cure is still a possibility or as long patients are not care-dependent. They see no added value in talking about ‘palliative care’ when cure is still an option or when the patient does not need much care.

GPs often do give emotional support around the diagnosis, but they do not want to designate this as palliative care. Some of them also indicated that the word ‘palliative’ in communication with a patient who is still living life to the full, is also not a very useful term.

### Strengths and limitations

So far, little empirical research had been conducted regarding the assumption that palliative care should start early in the course of the disease. Our research seeks to close this gap.

Most previous palliative care research has focused exclusively on patients with cancer [13-15]. A strength of this study is that it also focuses on GPs’ experiences with people with chronic non-cancerous conditions.

This study was based on qualitative interviews providing 'rich' detailed data on subjective experiences and views. Qualitative methods are recommended when research topics are relatively unexplored and no structured measurement instruments are available, as was the case in our study. However, qualitative designs have limitations regarding generalizability and external validity. Since a non-random purposive sample of Dutch GPs was involved in the study, it cannot automatically be concluded that this provides a representative picture of all GPs in the Netherlands. For instance, some overrepresentation can be expected of GPs with above-average interest in and experience with palliative care. Moreover, there may be limitations concerning the generalizability of the findings to other countries. Cross-national research shows that the Netherlands is known to be a country where doctors communicate relatively openly about end-of-life issues [16]. Therefore, it can be expected that patients in other countries will be very unlikely to communicate palliative care needs at an earlier stage than in this study.

### Implications for research and practice

Future quantitative research among a random sample of Dutch GPs as well as cross-national research is recommended to get a more complete and generalizable picture of how GPs recognize that a patient is in need of palliative care. It is recommended that future research should include both GPs with considerable experience of palliative care and GPs with less experience. In addition, it would be interesting to combine and compare multiple perspectives in a future study: those of the patients, the family members, the GPs and of other professional caregivers.

Finally, we recommend further research on the identification of palliative care needs in the ‘oldest old’ in particular, who often suffer from complex co-morbidity and show a steady decline up to death. Further research on this group of elderly people is important because this non-cancer group has not received much attention so far in palliative care research.

This study indicated that GPs prefer to avoid the term 'palliative' as long as they have not received signals from the patient that he/she is in need of palliative care or if curative treatments are still possible. Discussions are recommended on the use of the term 'palliative' or options for alternative terms, for instance in the training and education of healthcare professionals. The education and training of GPs could also include further discussion of what attitude GPs should take in palliative care. It is known from other recent research that Dutch GPs in general have a reactive rather than a proactive attitude in the interaction with their patients [17]. GPs assume that patients themselves should say what kind of support they need and what kind of problems they have. GPs do not want to patronise their patients or give care that is not needed. However, in general patients will not ask explicitly and clearly for palliative care in the early stages of the disease trajectory, which makes it even more important for GPs to initiate discussions on evolving care needs. A more proactive approach with the GP taking initiatives for advance care planning, may result in a better match with patients’ and family members’ existing and evolving care needs. We recommend further research into the conditions, stages and specific patient categories for which it would be better for GPs to opt for a proactive (rather than reactive) approach in patients with progressive diseases.

## Conclusion

GPs recognize a need for palliative care on the basis of various signals. They do not support the idea underlying Lynn and Adamson’s model that palliative care always starts early in the course of the disease.

## Competing interests

The authors declare that they have no competing interests.

## Authors’ contributions

SJJC and AF drafted this manuscript in cooperation with YE and LD. AF, YE and LD were responsible for the design of this study. SJJC and AF were involved in the data collection. SJJC, AF, YE and LD were involved in the analysis and/or interpretation of the data. All authors read and approved the manuscript.

## Pre-publication history

The pre-publication history for this paper can be accessed here:

http://www.biomedcentral.com/1471-2296/14/42/prepub

## Supplementary Material

Additional file 1 Topic list and interview questions. Click here for file
