# Supplementary material for: How do GPs identify a need for palliative care in their patients? An interview study
Source: BMC Fam Pract. 2013 Mar 25;14:42. doi: 10.1186/1471-2296-14-42 (PMC3617003; doi:10.1186/1471-2296-14-42)
Supplement: Additional file 1 — Topic list and interview questions. [file 1471-2296-14-42-S1.doc]

**Topic list and interview questions**

| Context |
| --- |
| • How would you define palliative care? What is the essential feature, in your opinion? |
| • Do you currently have patients who are receiving palliative care? |
| • Can you give a broad description of the patients you are currently giving palliative care to and the care you are giving them? |
| Start of the palliative phase |
| • What made you realize that these patients needed palliative care? |
| • Do you see a difference between cancer patients and non-cancer patients when it comes to recognizing a need for palliative care? |
| • How do you see your role as a GP if a cancer patient is still being treated by a medical specialist? |
| • What makes it easier, or more difficult, to recognize a need for palliative care? |
| • Does the point when you start palliative care often coincide with the prognosis that the patient has only a few more months to live? |
| If yes, why? If not, why not? |
| • Are there cases where you start palliative care many months, or even years, before death is expected? |
| Guiding principle for palliative care. |
| Nowadays, palliative care is often seen as more than simply terminal care. It is sometimes suggested that palliative care starts as soon as a life-threatening condition is diagnosed. What are your views on this? |
